# Development of fine motor skills is associated with expressive language outcomes in infants at high and low risk for autism spectrum disorder

**DOI:** 10.1186/s11689-018-9231-3

**Published:** 2018-04-12

**Authors:** Boin Choi, Kathryn A. Leech, Helen Tager-Flusberg, Charles A. Nelson

**Affiliations:** 1 Graduate School of Education, Harvard University, Cambridge, MA USA; 2 Graduate School of Arts and Sciences, Harvard University, Cambridge, MA USA; 3 Boston University School of Education, Boston, MA USA; 4 Department of Psychological and Brain Sciences, Boston University, Boston, MA USA; 5 Laboratories of Cognitive Neuroscience, Division of Developmental Medicine, Boston Children’s Hospital, Harvard Medical School, Boston, MA USA

**Keywords:** Autism, Fine motor skills, Expressive language, Early development, Infant siblings

## Abstract

**Background:**

A growing body of research suggests that fine motor abilities are associated with skills in a variety of domains in both typical and atypical development. In this study, we investigated developmental trajectories of fine motor skills between 6 and 24 months in relation to expressive language outcomes at 36 months in infants at high and low familial risk for autism spectrum disorder (ASD).

**Methods:**

Participants included 71 high-risk infants without ASD diagnoses, 30 high-risk infants later diagnosed with ASD, and 69 low-risk infants without ASD diagnoses. As part of a prospective, longitudinal study, fine motor skills were assessed at 6, 12, 18, and 24 months of age and expressive language outcomes at 36 months using the Mullen Scales of Early Learning. Diagnosis of ASD was determined at the infant’s last visit to the lab (18, 24, or 36 months) using the Autism Diagnostic Observation Schedule.

**Results:**

Hierarchical linear modeling revealed that high-risk infants who later developed ASD showed significantly slower growth in fine motor skills between 6 and 24 months, compared to their typically developing peers. In contrast to group differences in growth from age 6 months, cross-sectional group differences emerged only in the second year of life. Also, fine motor skills at 6 months predicted expressive language outcomes at 3 years of age.

**Conclusions:**

These results highlight the importance of utilizing longitudinal approaches in measuring early fine motor skills to reveal subtle group differences in infancy between ASD high-risk and low-risk infant populations and to predict their subsequent language outcomes.

**Electronic supplementary material:**

The online version of this article (10.1186/s11689-018-9231-3) contains supplementary material, which is available to authorized users.

## Background

Autism spectrum disorder (ASD) is characterized by deficits in social communication and interaction and repetitive and restricted behaviors [1]. While the hallmarks of ASD are impairments in social communication and interaction, a growing body of evidence suggests that the disorder is also associated with impaired motor development. For example, a meta-analysis reported that individuals with ASD show substantial impairments in motor coordination, compared with typically developing control participants [2]. A comprehensive review on motor functioning in ASD suggested that children and adults with ASD exhibit persistent difficulties across a wide set of motor behaviors including fine and gross motor skills and postural control [3].

Fine motor skills are one specific domain for which deficits and delays are common in ASD [3, 4]. These skills refer to one’s ability to make fine hand movements that often require sophisticated object manipulation and appear more vulnerable to delay in ASD relative to general gross motor behaviors such as walking [5]. In fact, children and adults with ASD show difficulties in fine motor skills ranging from grasping toys to handwriting [3]. Moreover, infants with an older sibling with ASD, who have an approximately 20% chance of developing the disorder themselves [6] (hereafter, “high-risk”), exhibit deficits and delays in fine motor skills in the first few years of life [4, 7–11]. A recent meta-analysis of 34 studies reported that high-risk infants as a group show significantly poorer fine motor skills measured on the Mullen Scales of Early Learning [12], compared to low-risk infants who do not have a family history of ASD [13]. Specifically, the study identified 12 months as the earliest point when differences in fine motor skills can be reliably detected between high- and low-risk groups. Relatedly, another study found that among high-risk infants, those who subsequently developed ASD exhibited more pronounced and persistent motor difficulties, relative to high-risk infants who were later typically developing [4].

Furthermore, a growing number of studies have suggested that motor abilities are associated with skills in other domains such as language in both typical and atypical development (for review, see [14, 15]). In children with ASD specifically, motor skills in the first 2 years predict expressive language at 4 years [16] and later speech fluency [17]. In high-risk infants, fine motor skills between 12 and 24 months significantly predict expressive language scores at 3 years [4]. And, more recently, early motor skills were found to be associated with the rate of expressive language development in high-risk infants who develop ASD [18]. These findings thus suggest that motor and language skills are interrelated in development.

One possible explanation for the relation between motor and language skills is that development of skills in one domain (i.e., motor) can extend across other domains (i.e., language) over time to influence an outcome—a concept known as *developmental cascades* [19]. Specifically, infants with new motor skills have new learning opportunities to interact with the environment and people, which may subsequently influence how others interact with them, which in turn facilitates child language development. For instance, a previous study found that 13-month-olds who could walk shared objects with their mothers more frequently than those who could only crawl [20]. Also, mothers of walking infants, in turn, were twice more likely to respond to their infants than mothers of crawling infants. Similarly, infants who can pick up objects such as a toy block are more likely to share it with their caregivers, who can then provide the label for the object (e.g., “do you want to build blocks?”). The response, in turn, helps the infant learn the word “block.” In short, a change in fine motor skills can alter how infants interact with objects and people, which may facilitate their language learning.

Given evidence of the motor-language links in development, deficits in early fine motor skills may help identify children who are likely to have language difficulties at a later age. Examining this possibility seems particularly relevant to infants at high risk for ASD who also have an increased prevalence of language and communication delays [21–23]. Identifying children at risk for future language difficulties would be useful so that targeted intervention programs can be made available to them in a timely fashion.

Despite the promising research benefits of studying early fine motor skills in ASD, there are several limitations to previous work that must be acknowledged. First, although subtle group differences in early fine motor skills at single time points have been noted, growth trajectories of fine motor skills in infants at high and low risk for ASD have yet to be thoroughly studied across infancy (see [7, 22] for notable exceptions). Studying how children’s fine motor skills develop over time may help depict a more complete picture of early development in infants at high and low risk for ASD than collecting a snapshot of their abilities at a single age. Relatedly, it remains unclear whether and to what extent growth trajectories of early fine motor skills are related to later language skills in infants at high and low risk for ASD. Previous research has pooled fine motor skill data across different time points (i.e., using composite scores of fine motor skills) to predict language outcomes (e.g., [4, 17]). Although useful, prior research thus leaves the open question of which specific growth parameters of fine motor skills (i.e., a child’s status, velocity, and acceleration in fine motor skills) may help predict subsequent language skills.

In the current study, we studied growth, or change over time, in fine motor skills between 6 and 24 months in relation to expressive language scores at 36 months in infants at high and low familial risk for ASD. By examining growth, we investigated whether groups differ in their trajectories of fine motor skills and determined which growth parameters of fine motor skills are linked to later language outcomes. First, we employed a unique growth modeling approach to ask whether growth in fine motor skills may differentiate three diagnostic groups: high-risk infants who were later diagnosed with ASD (HRA+), high-risk infants with no ASD diagnosis (HRA−), and low-risk control (LRC) infants with no diagnosis. We then used individual growth parameters of fine motor skills to predict expressive language at 36 months. Our specific research questions were as follows:

1. Do HRA+, HRA−, and LRC infants differ in their growth trajectories of fine motor skills between 6 and 24 months of age?
2. Do growth parameters of early fine motor skills (i.e., a child’s status, velocity, and acceleration in fine motor skills) predict expressive language at 36 months?

## Methods

### Participants

Participants were drawn from a prospective, longitudinal study of infants at high and low risk for ASD across the first 3 years of life. Eligibility criteria for all infants included a gestational age of at least 36 weeks, no known prenatal or perinatal complications, and no known genetic disorders. For the present study, the sample included infants who had fine motor skill data available for at least one time point at 6, 12, 18, and/or 24 months and an ASD evaluation at their last visit to the lab (either at 18, 24, or 36 months). The final analysis sample included 170 infants.

Of the 170 infants, 101 infants were classified as high risk for autism (HRA) because they had an older sibling with a community diagnosis of ASD. To verify older siblings’ ASD diagnoses, we used the Autism Diagnostic Observation Schedule (ADOS) [24] and/or age-appropriate screeners including the Social Communication Questionnaire (SCQ), for probands older than four, [25] and the Pervasive Developmental Disorders Screening Test-II (PDDST-II) [26], for probands younger than four, with the best clinical judgment by a psychologist, where required.

ASD diagnoses in 52 older siblings of HRA infants (51% of the HRA sample in the current study) were verified using both the ADOS and SCQ. Four HRA older siblings (4%) had the ADOS. Thirty-seven older siblings (37%) had their diagnosis verified using the SCQ, and three older siblings (3%) had the PDDST-II, as they did not have the ADOS. Five older siblings (5%) did not have an ADOS, SCQ, or PDDST-II and therefore were unable to have their diagnoses verified; however, all five of them had received their ASD diagnoses in specialist clinics, and data from their younger siblings were included in the current study.

Sixty-nine infants were classified as low-risk control infants (LRC) if they had a typically developing older sibling and no first- or second-degree family members with ASD. ASD diagnoses in 48 older siblings of LRC infants (70% of the LRC sample) were verified using both the ADOS and SCQ. Three older siblings (4%) had the ADOS. Thirteen older siblings (19%) had the SCQ and one sibling (1%) had the PDDST-II, as they did not have the ADOS. Finally, four LRC older siblings (6%) did not have an ADOS, SCQ, or PDDST-II; however, data from their younger siblings were included in the study, as their parents reported no clinical concerns in the older siblings.

For purposes of analyses, infants were further categorized into three groups based on their risk status (high- vs. low-risk) and an eventual ASD diagnosis (ASD vs. no ASD). Of the 101 HRA infants, 30 later met criteria for ASD (HRA+) and 71 did not meet criteria for ASD (HRA−). Of the 69 LRC infants, none met criteria for ASD (LRC).

Demographic characteristics for participants were collected at the first laboratory visit and are shown in Table 1, broken down by three groups and age. Infants in the three groups did not differ significantly on their race/ethnicity, sex distributions, and household income. However, there was a significant group difference in maternal education.^1^ For data analysis, a composite score for socioeconomic status (SES) was generated by combining household income and maternal education using principal component analysis, as the two variables were significantly and positively related to each other (*r* = .29, *p* = .0004). The first principal component weighted maternal education and income positively and equally and explained about 64% of the original variance (*M* = 0, *SD* = 1.14). The group difference remained significant in the SES composite such that HRA+ infants had the lowest level of SES.

**Table 1:** Sample characteristics, by age and group

	HRA+	HRA−	LRC	^*c*^ *p (*3-group)
Sex (% female)	30.0 *n* = 30	53.5 *n* = 71	44.9 *n* = 69	.09
Race/ethnicity (% White)	83.3 *n* = 30	95.7 *n* = 71	88.4 *n* = 69	.08
^a^ Household income	7.08 (2.02) *n* = 24	7.69 (0.91) *n* = 67	7.52 (1.38) *n* = 58	.79
^b^ Mother’s level of education	5.04 (1.72) *n* = 25	5.74 (1.65) *n* = 68	6.65 (1.22) *n* = 62	.0002^***^
Actual age at visits (month)				
6 months	5.91 (0.43) *n* = 22	5.96 (0.28) *n* = 50	5.97 (0.36) *n* = 61	.79
12 months	11.93 (0.45) *n* = 30	11.94 (0.38) *n* = 67	11.87 (0.42) *n* = 67	.54
18 months	18.12 (0.78) *n* = 25	17.91 (0.42) *n* = 67	18.01 (0.27) *n* = 67	.11
24 months	24.16 (0.55) *n* = 25	24.03 (0.55) *n* = 61	24.10 (0.56) *n* = 63	.60
36 months	36.09 (0.68) *n* = 22	36.57 (1.50) *n* = 51	36.33 (0.64) *n* = 54	.20

Data are reported as group means with standard deviations in parentheses

^a^Income was reported on an 8-point scale: (1) less than $15,000, (2) $15,000–$25,000, (3) $25,000–$35,000, (4) $35,000–$45,000, (5) $45,000–$55,000, (6) $55,000–$65,000, (7) $65,000–$75,000, (8) more than $75,000

^b^Education was reported as the highest level attained on a 9-point scale: (1) some high school, (2) high school graduate, (3) some college, (4) community college/two-year degree, (5) four-year college degree, (6) some graduate school, (7) master’s degree, (8) doctoral degree, (9) professional degree

^c^Fisher’s exact tests were used to determine *p* values for group differences in sex and race/ethnicity. Kruskal-Wallis tests were used to determine *p* values for group differences in income and maternal education. One-way ANOVA tests were used to determine *p* values for group differences in age

***p < .001

### Procedures

This study was approved by the Institutional Review Boards (IRBs) at Boston Children’s Hospital and Boston University. Written, informed consent was obtained from all caregivers prior to their infants’ participation in the study. Infants were recruited and allowed to enter the study at different ages (e.g., 6 or 12 months) as long as their first visit took place no later than 12 months of age.

At 6, 12, 18, 24, and 36 months of age, trained examiners administered the Mullen Scales of Early Learning (MSEL) [12] to children who visited the laboratory*.* ASD diagnoses were made at 18, 24, and 36 months. At the child’s last visit (either 18, 24, or 36 months), final ASD diagnoses for children were determined on the basis of the ADOS using the revised algorithm, with the best clinical judgment by a psychologist, where required. If there were multiple diagnostic evaluations (e.g., children completed ASD evaluations at 18, 24, and 36 months), the ultimate categorization was made at the last visit (e.g., 36 months) by a licensed psychologist. Depending on the child’s last visit, ASD outcome classifications were made at 18 months for 13 children (8% of the sample in the present study; *n*_HRA+_ *=* 3; *n*_HRA−_ *=* 4; *n*_LRC_ *=* 6), at 24 months for 24 children (14%; *n*_HRA+_ *=* 3; *n*_HRA−_ *=* 13; *n*_LRC_ *=* 8), or at 36 months for 133 children (78%; *n*_HRA+_ *=* 24; *n*_HRA−_ *=* 54; *n*_LRC_ *=* 55). Although the majority of our children had their ASD outcome classifications made at 36 months, the rest of the children had their ASD outcomes made at earlier age points (18 or 24 months) due to sample attrition. As prior research suggests the high diagnostic stability of ASD at 18 and 24 months [27, 28], infants with ASD diagnoses made between 18 and 36 months were included in the current study, similar to previous studies [29, 30].

### Measures

#### Mullen Scales of Early Learning (MSEL) [12]

The MSEL is a standardized, normed, developmental assessment for children from 0 to 68 months and provides an overall index of cognitive ability and potential delay. The MSEL consists of five scales: Gross Motor, Visual Reception, Fine Motor, Expressive Language, and Receptive Language. In this study, we used the MSEL Fine Motor, Expressive Language, and Visual Reception scales. More specifically, we used raw scores from the Fine Motor scale at 6, 12, 18, and 24 months to study longitudinal trajectories of children’s fine motor skills development. We used children’s raw scores from the Expressive Language scale at 36 months to assess children’s expressive language outcomes. Raw scores from the Visual Reception scale at 6 months were used as a covariate to control for nonverbal cognition in regression analyses of fine motor and expressive language relations, as variation in children’s early fine motor skills may arise from differences in general nonverbal skill [4]. The possible ranges of raw scores for the Fine Motor scale are 0 to 49 and 0 to 50 for the Expressive Language and Visual Reception scales. We used raw scores rather than standardized scores (i.e., *T* scores), as raw scores allowed us to better capture individual differences in skills across time. Relatedly, we used child exact age at each visit as our measure of time to control for differences in age of testing (Table 1), which might affect children’s raw scores on MSEL.

#### Autism Diagnostic Observation Schedule (ADOS) [24]

The ADOS is a semi-structured play assessment of social interaction, communication, and restricted interests/repetitive behavior. Research staff with extensive experience in testing children with developmental disorders administered and scored children’s ADOS. In addition, an ADOS-reliable researcher co-scored the ADOS via video recording. When children met the criteria for ASD on the ADOS or came within three points of cutoffs, a licensed clinical psychologist reviewed the ADOS scores and behavioral assessment videos to determine final clinical judgment: ASD or no ASD.

### Data reduction and analysis

Because of data attrition associated with longitudinal design (e.g., infants not yet enrolled in study, visits missed by families), 6-month fine motor skill data were available for *n* = 133, 12-month data for *n* = 164, 18-month data for *n* = 159, and 24-month data for *n* = 149 children. Of note, 110 of 170 infants contributed fine motor skills data at all four time points.

In order to address our research goals, we carried out analyses in two stages. In the first step, to explore group differences, we used hierarchical linear modeling (HLM) to best characterize each group’s fine motor skills growth between 6 and 24 months (see Additional file 1). HLM allowed us to model developmental trajectories of each individual and accommodate the nested, hierarchical nature of the data (i.e., multiple measurements within infants) and missing data in our longitudinal design. Applications of HLM for growth involved a two-level hierarchical structure, where we first modeled each child’s change over time in fine motor skills (Level 1) and then determined whether fine motor skills among the groups showed differences in growth parameters (Level 2). Specifically, at Level 1 (within children), we included time-variant predictors such as a linear age variable (*age)* and a quadratic age variable (*age*^*2*^*).* We centered age at the earliest data collection point, 6 months or 0.5 years, so that parameters are more interpretable [31] and reflect children’s fine motor skills and rate of growth at 6 months. In addition, we performed post hoc analyses by re-centering time so that the trajectories’ intercept systematically varied by age (i.e., *age*_*ti*_–12, *age*_*ti*_–18, *age*_*ti*_–24). Re-centering time allowed us to examine the point at which the divergence of developmental trajectories between outcome groups became statistically significant. If we did not center age, the model would estimate growth rates when children are at birth (i.e., 0 months), for which we would expect no measureable fine motor skill or growth. The quadratic age variable (*age*^*2*^*)* represents the acceleration (or deceleration) in the rate of change and was calculated by squaring the centered linear age variable. At Level 2 (between children), time-invariant predictors included groups (*group*; HRA+, HRA−, LRC). The fully specified equation for our model is summarized in Additional file 1.

In the second step, our goal was to determine which growth parameters of early fine motor skills (i.e., status, velocity, and acceleration) between 6 and 24 months explain significant variance in children’s expressive language outcomes at 36 months. Thus, we employed individual growth rates of fine motor skills from our Level-1 HLM model as independent variables to predict later expressive language skills in regression analyses (see Additional file 2). That is, we used a prediction model, in which we calculated individual growth rates, or Empirical Bayes’ posterior means [32], using the random effects and fixed effects coefficients from our HLM model that includes only Level 1 predictors. An estimate was created for each child computed from a weighted combination of the individual child’s growth trajectory (the random effect coefficient) as well as the average trajectory of the entire sample (the fixed effect coefficient). The rationale for using the method of Empirical Bayes stems from prior work that shows these Empirical Bayes’ predictions from models are unbiased and precise (i.e., more similar to true values) than the predictions generated from a standard ordinary least squares (OLS) regression [32]. Similar to previous work employing the same analytic strategy [33], we found that the three predictors were too collinear to include simultaneously into one regression model. Thus, we fit three separate models for each predictor. All analyses were conducted using Stata and R, and HLM models were fit with the lmer package within R [34].

## Results

### Modeling fine motor skills growth

Descriptive data on cross-sectional fine motor skills, as measured on MSEL, between 6 and 24 months are presented in Table 2. Of note, although HRA+ infants as a group demonstrated lower raw scores on the MSEL Fine Motor scale, compared to HRA− and LRC infants, the scores of all groups were within the range of typical development.

**Table 2:** Means, standard deviations, ranges, and sample sizes of cross-sectional data from the MSEL Fine Motor scale, by age and group

Age	HRA+	HRA−	LRC	*p* (3-group)	*d (HRA+* vs. *HRA*−*)*	*d (HRA+* vs. *LRC)*	*d (HRA*− vs. *LRC)*
6 months	7.86 (1.21) 6–11 *n* = 22	8.42 (1.28) 6–12 *n* = 50	8.13 (1.27) 6–12 *n* = 61	0.20	− 0.44	− 0.22	0.23
12 months	16.5 (2.08) 12–21 *n* = 30	17.27 (1.80) 12–20 *n* = 67	16.58 (1.63) 12–20 *n* = 67	0.04	− 0.41	− 0.05	0.40
18 months	20.36 (1.66) 16–24 *n* = 25	21.03 (1.64) 17–24 *n* = 67	20.96 (1.54) 19–25 *n* = 67	0.19	−0.41	− 0.38	0.04
24 months	24.12 (2.42) 19–28 *n* = 25	25.13 (2.12) 20–29 *n* = 61	25.56 (2.59) 20–32 *n* = 63	0.04	−0.46	− 0.57	−0.18

To best characterize the developmental trajectories of fine motor skills between 6 and 24 months in HRA+, HRA−, and LRC infants, we used the following model building strategies. Preliminary visual inspection of the raw data suggested fine motor skills followed a curvilinear trajectory between 6 and 24 months. Statistical analyses confirmed this pattern: the best fitting model to the data contained both a linear and quadratic growth term, *− 2 Log Likelihood* = − 1231, *χ*^*2*^ (7) = 1481, *p* < .001. Next, we added the interaction between group (HRA+, HRA−, LRC) and age (both linear and quadratic) to determine whether change in fine motor skills differed between groups. Results revealed that the groups differed significantly from one another in the linear growth only, $\widehat{\beta}$_HRA+ × AGE_ = − 0.83, *SE*_HRA+ × AGE_ = 0.39, *p* = .04; $\widehat{\beta}$_HRA− × AGE_ = − 0.51, *SE*_HRA− × AGE_ = 0.30, *p* = .09. A model with a *group × quadratic age* interaction fit the data no better than a model without it. As such, we removed this term and retained only the *group × linear age* interaction in subsequent models. We completed our model building process by adding demographic covariates (i.e., sex and SES). Neither of the covariates significantly predicted fine motor skills and were thus not included in the final model.

Our final HLM model summaries are presented in Table 3. The final model considered between-child associations of groups with status (intercept), velocity (linear growth), and acceleration (quadratic growth) in fine motor skills and an interaction effect between groups and linear growth at 6 months. Note that we entered the LRC group into all models as a reference group; therefore, the coefficients generated for HRA+ and HRA− groups reflected deviations in intercept, slope, and acceleration from the LRC group. The final model shows that, on average, LRC infants at 6 months had estimated fine motor skills of approximately 8 points, with an increase in fine motor skills at this age at a rate of 17.71 points per year. Of note, after studying how high-risk infants differed in their fine motor skills development from those of low-risk infants (i.e., LRC as a reference group), we systematically rotated which group served as the comparison to examine potential differences in growth trajectories among three groups.

**Table 3:** Final growth model of group predicting growth trajectories for fine motor skills (age centered at 6 months; *N* = 170)

	Coefficient	SE
Intercept	8.38^***^	0.19
Linear growth	17.71^***^	0.44
Quadratic growth	−4.38^***^	0.27
HRA+	0.08	0.34
HRA−	0.58^*^	0.26
Linear × HRA+	−0.83^*^	0.39
Linear × HRA−	−0.51	0.30
Variance components		
Goodness of fit (− 2 Log Likelihood)	− 1226.94	
Variance in intercept	0.27	
Variance in growth rate	1.70	
Variance in acceleration	1.72	

^***^p < .001, ^*^p < .05

**Fig. 1:**
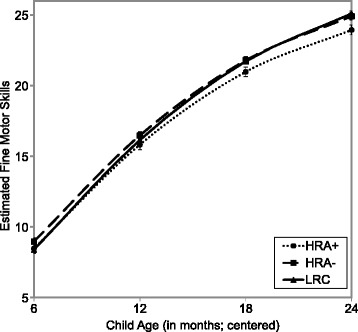
The average growth in fine motor skills for three groups. Error bars represent the standard error of the mean

Estimated growth trajectories of fine motor skills from 6 to 24 months are presented for all three groups in Fig. 1.

As can be seen in the figure, when we compared *status* of fine motor skills (i.e., intercept) among three groups, HRA+ infants did not significantly differ from their typically developing peers (both HRA− and LRC) at 6 months, indicating that the three groups were indistinguishable by their fine motor skills at 6 months. However, when age was re-centered at 12, 18, and 24 months to identify points of divergence in developmental trajectories of fine motor skills, HRA+ infants showed significantly lower fine motor skills than HRA− infants starting at 12 months (*t* = 2.45, *p* = .015) and LRC infants at 18 months (*t* = − 2.34, *p* = .02). Thus, these results indicate that infants later diagnosed with ASD began to diverge from their typically developing peers by their first birthday, although when these groups diverged depended on whether they were infants at high or low risk for autism. Interestingly, at 6 months, HRA− infants had significantly stronger fine motor scores compared to LRC infants, *t* = 2.19, *p* = .03, but this difference no longer reached significance beginning at 12 months.

When we compared *velocity* in fine motor skills (i.e., linear growth) among the three groups, HRA+ infants had significantly slower growth rates than LRC infants at 6 through 24 months, *t* = − 2.11, *p* = .036. Specifically, the average growth rate for the HRA+ group (*M* = 16.88, *SE* = 0.39) was approximately two standard errors below the mean of the LRC group (*M* = 17.71, *SE* = 0.44). The HRA− infants also had slower growth rates than the LRC group between 6 and 24 months, but this difference did not reach statistical significance, *t* = − 1.68, *p* = .094. Thus, while the groups demonstrated comparable status of fine motor skills at 6 months, HLM revealed subtle group differences in growth in fine motor skills from 6 months.

### Using growth parameters of fine motor skills to predict expressive language outcomes

To investigate which growth parameters of fine motor skills between 6 and 24 months predict expressive language outcomes at 36 months, we first examined descriptive statistics on the language outcomes (Table 4) and found significant group differences on 36-month expressive language scores. Specifically, high-risk infants scored significantly lower on the MSEL Expressive Language scale at 36 months, compared to low-risk infants.

**Table 4:** Means and standard deviations for MSEL Expressive Language scale raw scores at 36 months and MSEL Visual Reception scale raw scores at 6 months

	HRA+	HRA−	LRC	*p (*3-group)
MSEL Expressive Language at 36 months	31.32 (4.28) *n* = 22	35.02 (4.53) *n* = 51	37.26 (3.89) *n* = 54	< .0001^***^
MSEL Visual Reception at 6 months	8.41 (1.74) *n* = 22	8.6 (1.54) *n* = 50	8.44 (1.65) *n* = 61	.85

^***^p < .001

Next, we fitted a series of regression models with each of the growth parameters as the independent variable and expressive language scores as the dependent variable. In regression analyses, we controlled for children’s nonverbal cognition, as indexed by MSEL visual reception scores at 6 months, to evaluate whether variance in expressive language skills was accounted for by the fine motor growth parameters above and beyond any variance accounted for by children’s general nonverbal skill. The visual reception scores, assessed independently of children’s motor abilities, did not differ across groups at 6 months (Table 4). In addition, child sex and SES, which significantly differed across groups and also are identified as related to children’s language skills in previous research [35, 36], were included as covariates.

Table 5 shows the results of regression analyses. Specifically, the status of fine motor skills at 6 months (Model 1) was a significant, positive predictor of 36-month expressive language scores, when controlling for 6-month visual reception scores, sex, and SES. In other words, these results illustrate that when accounting for children’s general nonverbal cognition, sex, and SES, a child whose 6-month fine motor skills scored at the sample mean had an expressive language score of 35 points at 36 months, and every one unit increment in fine motor skills at 6 months was associated with approximately four-point difference (*SE* = 1.22) in 36-month language scores, *t* = 3.64, *p* < .001, *R*^2^ = 23.6%, 95% CI = [2.02, 6.88]. When accounting for the covariates, the velocity in fine motor skills (Model 2) was marginally significant in predicting later expressive language scores, *t* = 1.81, *p* = .07, *R*^2^ = 14.4%. The acceleration (Model 3) did not result in significant variance explained in later language skills, *t* = − .03, *p* = .98, *R*^2^ = 10.9%. As the models were not nested, we compared Bayesian Information Criterion (BIC) estimates to determine which growth parameter best accounted for variance in the language outcomes. This comparison suggests that the model with the status of fine motor skills had the lowest BIC (510) value, relative to the models with the velocity or acceleration parameters (*BICs* = 520, 523, respectively). Altogether, a child’s 6-month fine motor skills provided the most helpful information to estimate the child’s expressive language at 3 years of age.

**Table 5:** Growth models predicting 36-month Expressive Language skills, when controlling for nonverbal cognition, SES, and sex

	Model 1	Model 2	Model 3
Intercept	34.89^***^	33.28^***^	31.82^***^
Predicted status at 6 months	4.45^***^		
Predicted velocity at 6 months		1.14~	
Predicted acceleration at 6 months			−0.02
Nonverbal cognition at 6 months	0.07	0.25	0.39
SES	1.45^**^	1.54^**^	1.50^**^
Sex	0.08	0.63	0.97
*R* ^2^	23.6%	14.4%	10.9%
BIC	510	520	523

^***^p < .001, ^**^p < .01

Finally, we included interaction terms between each of the growth parameters and groups (e.g., *linear x group*) to determine whether the relations between growth parameters of fine motor skills and expressive language outcomes differ across groups. A model resulted in no significant interactions between growth parameters and groups, suggesting that the effect of fine motor skills on later expressive language outcomes did not differ for HRA+, HRA−, and LRC infants.

## Discussion

In the current study, we examined growth trajectories of fine motor skills between 6 and 24 months and determined which growth parameters of fine motor skills predict language outcomes at 36 months in high-risk infants later diagnosed with ASD, high-risk infants with no ASD diagnosis, and low-risk infants with no ASD diagnosis. Our key findings were that the development of fine motor skills was slower between 6 and 24 months in high-risk infants later diagnosed with ASD, compared to that of their typically developing peers, and that early fine motor skills were associated with subsequent expressive language skills at 36 months in all three groups.

### Growth trajectories of early fine motor skills

HLM revealed that infants at high risk for ASD who themselves later developed ASD had slower *growth* in fine motor skills between 6 and 24 months of age, compared to infants at low risk for ASD. This finding is consistent with those of previous studies that also employed longitudinal approaches and examined infants’ performance on the MSEL. Specifically, a prior study reported that high-risk infants later diagnosed with ASD deviated from unaffected infants at around 14 months and developed more slowly through 24 months on the MSEL Fine Motor scale [8]. Similarly, another study using latent class analysis identified slower developmental trajectories of fine motor skills in children with ASD between 6 and 36 months, compared to children without ASD [37]. This study thus adds to the existing research suggesting slower development in infancy in children with ASD. However, while these group differences between high-risk infants with ASD diagnoses and low-risk infants without ASD diagnoses were statistically significant, they were subtle and small (Fig. 1; Table 2). Moreover, fine motor scores for all groups were within the range of typical development, indicating that these modest group differences may not rise to the level of detection by parents or clinicians in many cases.

Our data indicated that although high-risk infants later diagnosed with ASD showed slower growth in fine motor skills between the 6- and 24-month periods, relative to that of high-risk infants without ASD diagnoses, this difference was not statistically significant. This nonsignificant difference between the two high-risk groups suggests that slower fine motor growth may not be specific to ASD. Our finding is consistent with those from prior research indicating that fine motor differences may be a characteristic of infants at high risk for ASD, rather than a core characteristic of the disorder [10].

With regard to the *status* of fine motor skills at 6 months, there was no statistically significant difference between high-risk infants who later developed ASD and typically developing high- and low-risk infants. Only beginning in the second year of life, did high-risk infants who were later diagnosed with ASD score significantly lower on the MSEL Fine Motor scale than high- and low-risk infants without eventual diagnosis. The nonsignificant group difference in status of fine motor skills at 6 months stands in contrast to some of the prior findings that reported high-risk infants tend to show differences in fine motor skills, relative to their low-risk peers as early as 6 months [10]. Given mixed evidence of fine motor differences in infancy (i.e., 6–7 months), future research is needed to replicate the examination of fine motor skill development with larger samples, particularly within the first year of life in ASD risk populations.

To our surprise, high-risk infants who did not develop ASD showed stronger fine motor scores than low-risk infants at 6 months, but the difference was transient, with these infants showing comparable fine motor scores from 12 months onward. This difference may reflect a random sampling error. Alternatively, strong early fine motor skills may function as a protective factor, rather than a risk factor, for some high-risk infants. That is, while all high-risk infants presumably carry genetic risk factors for ASD, those with stronger fine motor skills may require greater familial etiologic load to manifest the ASD phenotype [38]. Examining the extent to which fine motor skills may act as a protective or risk factor for high-risk infants will be an important avenue for future research.

The results of the first part of our study highlight the importance of investigating the course of developmental change in skills over time. Studies of other behavioral domains lend support for this need to focus on developmental change [39]. For example, an eye-tracking study reported that infants later diagnosed with ASD showed a decline in fixation to the eye region of the face from 2 to 6 months and were distinguishable from their typically developing peers by change over time; however, cross-sectional group differences in eye fixation emerged only later in the first year [40]. Thus, while cross-sectional research identifies group differences at individual time points, longitudinal approaches can capture developmental change over time and depict a more complete and nuanced picture of early development in infants at high and low risk for ASD.

### Fine motor growth trajectories predict expressive language outcomes

In our analysis to determine which growth parameters of early fine motor skills predict subsequent expressive language outcomes at 36 months, we found that the status of fine motor skills from the 6- to 24-month growth model was a significant, positive predictor of later expressive language outcomes, even after controlling for nonverbal cognition scores, sex, and SES. In other words, infants with poorer fine motor skills across the first 2 years of life scored significantly lower on expressive language at 36 months, even when the covariates were taken into account. On the other hand, the velocity in fine motor skills was marginally associated with subsequent expressive language outcomes, and the acceleration was not significantly associated with the outcomes, when controlling for the covariates. Thus, it appears that status of early fine motor skills may provide the most useful information about later expressive language skills among the growth parameters (i.e., status, velocity, and acceleration).

Also, the significant, positive associations between early fine motor skills and subsequent expressive language outcomes did not differ across all three groups, suggesting that differences in fine motor skills over time can have cascading effects on language outcomes for both high-risk infants who later developed ASD and typically developing high- and low-risk infants. These findings align with prior work on developmental motor-language cascades [4, 41] demonstrating that children’s early motor skills are significantly and positively related to later expressive language skills in both typical and atypical development.

Finally, the associations between early fine motor skills and later language abilities highlight a potential avenue for early intervention practices. Given that the findings of this study suggest that fine motor skills in infancy may influence subsequent expressive language outcomes, an assessment of early fine motor skills holds promise for early identification of difficulties in language which emerge later in life in high-risk infants [21, 22]. By identifying and addressing infants’ difficulties in fine motor skills in a timely fashion, we may then prevent cascading effects of motor impairments on children’s language development. In fact, a growing body of literature suggests promising effects of early motor training on other domains of development. For example, “sticky mittens” with Velcro strips are associated with increased object exploration behaviors in infants that are shown positively related to subsequent language development [42, 43].

Our findings should be interpreted in light of key limitations, however. First, due to the high levels of maternal education, our sample may not be a nationally representative sample of infants at high and low risk for ASD. Therefore, findings may not be generalizable to the larger population of infants at high and low risk for ASD. Second, our study focused on examining the relations between early fine motor skill development and later expressive language outcomes in infants at risk for ASD. More studies are needed to closely investigate the motor-language relations in other neurodevelopmental disorders such as developmental language disorders and dyslexia. Third, ASD outcomes of 22% of our participants were made at 18 or 24 months instead of at 36 months, when diagnosis can be reliably made [44]. Therefore, it is possible that those diagnosed at 18 or 24 months would or would not have met criteria for ASD at 36 months. However, the best clinical judgment was made by an expert clinician for those children using comprehensive data including developmental history and standardized tools. In addition, recent studies suggest high diagnostic stability for infants at high familial risk at this age [27, 28]. While we made a decision to include those with ASD outcomes made at 18–36 months to maximize our sample size, future research could minimize the variation in age of diagnosis until there is more evidence for stable diagnosis as early as 18 months of age. Despite these limitations, our findings have the potential to promote longitudinal examinations of infants at increased risk for ASD and influence how we intervene to promote their optimal language outcomes.

## Conclusions

Overall, our results suggest that fine motor skills growth between 6 and 24 months is significantly slower in high-risk infants with eventual ASD diagnosis, compared to high- and low-risk peers without eventual diagnosis, and predicts expressive language skills at 3 years of age. This work highlights the importance of studying children’s skills within the context of developmental trajectories. Specifically, examining children’s developmental change over time may create a more complete picture than collecting a snapshot of their abilities at a single age. Finally, poor performance on early fine motor skills may indicate an increased risk for language difficulties in children and be addressed early in life to promote children’s optimal language outcomes. Targeting early fine motor skills in infancy seems promising, considering that these skills seem amenable to intervention [42, 43] and that children can have the most gains during sensitive periods when their brains are receptive to the environment [45]. Altogether, our results suggest that closer attention to developmental trajectories of fine motor skills in relation to later developmental outcomes may be warranted in infants at high familial risk for ASD.

## Additional files

Additional file 1: Equation for the final HLM model. (DOCX 32 kb)

Additional file 2: Equation for the linear model predicting 36-month expressive language. (DOCX 17 kb)

## Abbreviations

ADOS: Autism Diagnostic Observation Schedule
ASD: Autism spectrum disorder
HLM: Hierarchical linear modeling
HRA: High risk for autism
HRA +: High-risk infants with autism
HRA−: High-risk infants without autism
IRB: Institutional Review Board
LRC: Low-risk control
MSEL: Mullen Scales of Early Learning
OLS: Ordinary least squares
PDDST-II: Pervasive Developmental Disorders Screening Test-II
SCQ: Social Communication Questionnaire
SES: Socioeconomic status

## Footnotes

1 LRC mothers demonstrated the highest levels of maternal education. Although our sample as a whole was recruited from a relatively high socioeconomic area of greater New England area, our LRC families, in particular, had unusually high levels of maternal education. Specifically, the majority of mothers of LRC infants (60 out of 62 families; 97%) had at least four-year college degree, and the remaining two families (3%) indicated community college/two-year degree as the highest education level attained.
