# Supplementary material for: Development of fine motor skills is associated with expressive language outcomes in infants at high and low risk for autism spectrum disorder
Source: J Neurodev Disord. 2018 Apr 12;10:14. doi: 10.1186/s11689-018-9231-3 (PMC5898056; doi:10.1186/s11689-018-9231-3)
Supplement: Supplementary file 1 — Equation for the final HLM model. (DOCX 32 kb) [file 11689_2018_9231_MOESM1_ESM.docx]

**Additional file 1.** Equation for the final HLM model

**Level 1:**

$Y_{ti}= \pi_{0i}+ \pi_{1i}\left( {age}_{ti}-6 \right)+\pi_{2i}\left( {age}_{ti}-6 \right)^{2}+e_{ti},$ $e_{ti}\sim N(0,\sigma_{t}^{2})$

**Level 2:**

$$\pi_{0i}= \gamma_{00}{+\gamma_{01}\left( {group}_{i} \right)+u}_{0i}$$

$$\pi_{1i}= \gamma_{10}{+\gamma_{11}\left( {group}_{i} \right)+u}_{1i}$$

$$\pi_{2i}= \gamma_{20}{+ u}_{2i}$$

**

In the Level 1 equation, the growth parameter $\pi_{0i}$ represents the predicted level of fine motor skills of child *I* at 6 months. The parameter $\pi_{1i}$ represents the predicted linear rate of fine motor growth for child *i.* The parameter $\pi_{2i}$ represents the non-linear acceleration/deceleration in fine motor growth for each child over time. As is standard with these types of models, we assume an error structure where each$e_{ti}$ is normally distributed with mean of zero and variance $\sigma_{t}^{2}.$ In the Level 2 equation, $\pi_{0i}$, $\pi_{1i}$, and $\pi_{2i}$ represent the growth parameters from the Level 1 model. $\gamma_{p0}$ , $\gamma_{p1}$and $\gamma_{p2}$are linear regression coefficients. The coefficient on the term *group* is of main interest to the study. Finally, $u_{0i}$, $u_{1i}$, and $u_{2i}$ represent random effects, each with a mean of 0. The set of three random effects for child *i* are assumed multivariate normally distributed with full covariance matrix, T, dimensioned a 3x3 matrix.

$$T= \left[ \begin{matrix} \tau_{00} & & \\ \tau_{10} & \tau_{11} & \\ \tau_{20} & \tau_{21} & \tau_{22} \end{matrix} \right]= \left[ \begin{matrix} Var(\pi_{0i}) & & \\ Cov(\pi_{1i}, \pi_{0i}) & Var(\pi_{1i}) & \\ Cov(\pi_{2i}, \pi_{0i}) & Cov(\pi_{2i}, \pi_{1i}) & Var(\pi_{2i}) \end{matrix} \right]$$
