# Supplementary material for: Development of fine motor skills is associated with expressive language outcomes in infants at high and low risk for autism spectrum disorder
Source: J Neurodev Disord. 2018 Apr 12;10:14. doi: 10.1186/s11689-018-9231-3 (PMC5898056; doi:10.1186/s11689-018-9231-3)
Supplement: Supplementary file 2 — Equation for the linear model predicting 36-month expressive language. (DOCX 17 kb) [file 11689_2018_9231_MOESM2_ESM.docx]

**Additional file 2.** Equation for the linear model predicting 36-month expressive language

Similar to the methodological approach used by Rowe, Raudenbush, and Goldin-Meadow (2012), we assumed a linear prediction model for later expressive language as:

$$E\left( W_{i} | \pi_{0i}, \pi_{1i},\pi_{2i} \right)=\alpha+\gamma_{0}\pi_{0i}+ \gamma_{1}\pi_{1i}+\gamma_{2}\pi_{2i}+y_{3}X_{i}$$

- $W_{i}$ represents expressive language skills at 36 months
- $\gamma_{0}$, $\gamma_{1}$and $\gamma_{2}$ tells us how child *i*’s predicted fine motor skills (status, velocity, and acceleration) at age 6 months, that is, how $\pi_{0i}$, $\pi_{1i}$, and $\pi_{2i},$ contributes to $W_{i}$, controlling for background covariate $X_{i}$ (i.e., MSEL Visual Reception scores, sex, and SES).

The equation above represents our hypothesis that a child’s status, velocity and acceleration in fine motor skills at age 6 months help us predict 36-month expressive language, $W_{i}$, controlling for nonverbal cognition, sex, and SES. Since we cannot directly observe $\pi_{0i}, \pi_{1i,}$and $\pi_{2i}$, we estimated these unknowns using the Empirical Bayes’ predictions from the models to determine how expressive language changes as a function of these estimates.
